# Blood Toxic Elements and Effects on Plasma Vitamins and Carotenoids in Two Wild Bird Species: *Turdus merula* and *Columba livia*

**DOI:** 10.3390/toxics9090219

**Published:** 2021-09-11

**Authors:** Pablo Sánchez-Virosta, José Manuel Zamora-Marín, Mario León-Ortega, Pedro J. Jiménez, Silvia Rivas, Lidia Sánchez-Morales, Pablo R. Camarero, Rafael Mateo, Manuel Zumbado, Octavio P. Luzardo, Tapio Eeva, Antonio J. García-Fernández, Silvia Espín

**Affiliations:** 1 Area of Toxicology, Department of Socio-Sanitary Sciences, Campus de Espinardo, University of Murcia, 30100 Murcia, Spain; pjjm@um.es (P.J.J.); silvia.rivasg@um.es (S.R.); lidia.sanchezm1@um.es (L.S.-M.); ajgf@um.es (A.J.G.-F.); 2 Department of Zoology and Physical Anthropology, Faculty of Biology, University of Murcia, 30100 Murcia, Spain; josemanuel.zamora@um.es; 3 Grupo de Anillamiento ANSE, 30002 Murcia, Spain; 4 Asociación Ulula, 30110 Murcia, Spain; mlortega@um.es; 5 Instituto de Investigación en Recursos Cinegéticos, IREC (CSIC, UCLM, JCCM), Ronda de Toledo 12, 13005 Ciudad Real, Spain; PabloR.Camarero@uclm.es (P.R.C.); Rafael.mateo@uclm.es (R.M.); 6 Toxicology Unit, Research Institute of Biomedical and Health Sciences, University of Las Palmas de Gran Canaria, 35016 Las Palmas, Spain; manuel.zumbado@ulpgc.es (M.Z.); octavio.perez@ulpgc.es (O.P.L.); 7 Spanish Biomedical Research Center in Physiopathology of Obesity and Nutrition (CIBERObn), Paseo Blas Cabrera Felipe s/n, 35016 Las Palmas, Spain; 8 Department of Biology, University of Turku, FI-20014 Turku, Finland; teeva@utu.fi

**Keywords:** metal exposure, biochemistry, industrial emissions, urbanization, wild pigeon, blackbird

## Abstract

Birds have historically suffered adverse effects by toxic elements, such as As, Pb, Hg, and Cd. However, reports on exposure to a wide range of elements, including rare earth elements and other minor elements of emerging concern, and the potential consequences for wildlife are still scarce. This study evaluates blood concentrations of 50 elements and their related effects on lutein and vitamin levels in the Eurasian blackbird (*Turdus merula*) and wild rock pigeon (*Columba livia*), inhabiting different scenarios of contaminant exposure. Blood concentrations of As, Cd, and Pb (and Mn in *T. merula*) were increased in both species captured in the mining area, compared to the control site. *T. merula* also showed increased As, Cd, and Pb concentrations in blood in the agricultural–urban area, as compared to the control area, together with the highest Hg levels, which could be related to agricultural practices and industrial activities. Decreases of 33 and 38% in the plasma retinol levels in *T. merula* inhabiting the mining and the agricultural–urban areas, respectively, as compared to the control site, were associated with increased Pb, As, and Cd exposure. This could be due to a metal-driven suppressive effect in retinol metabolism and/or its over-use for coping with metal-related oxidative stress.

## 1. Introduction

Avifauna is a group of vertebrates that have historically suffered the adverse effects of contaminants [1,2,3,4,5]. Metalloids and metals including arsenic (As), lead (Pb), mercury (Hg), and cadmium (Cd) are known to be toxic and have been ranked in the first positions within the Substance Priority List by the Agency of Toxic Substances and Disease Registry (ATSDR) [6]. These toxic elements are extensively reported in tissues of different bird species, due to their bioaccumulation capacity and the negative effects they may cause, including alterations in growth, reproductive success, behavior, immune function, and biochemistry [2,4,7,8,9,10,11,12,13]. In addition to the metals/metalloids classically studied, rare earth elements (REE) and other minor elements (ME) are of emerging concern because of their use in modern technology worldwide, leading to emissions and electronic waste [14,15]. However, reports on exposure to these elements and the potential consequences for wildlife populations have only recently been studied and are still scarce [16,17,18]. Therefore, additional research is needed to evaluate toxic elements exposure, spatiotemporal trends, and related adverse effects for different avian species.

Vitamins and carotenoids are dietary nutrients with a wide range of physiological functions in the antioxidant and immune systems, vision, reproduction, and growth [5]. Carotenoids are essential for immune function, coloration, and breeding; some carotenoids are precursors of vitamin A [19] and their role as antioxidants is still under debate [20,21]. Retinol is the active form of vitamin A and plays critical roles in antioxidant and immune functions, as well as cell differentiation, proliferation, and growth [5,22,23]. Regarding α-Tocopherol, it is the major form of vitamin E and has a potent role as antioxidant, protecting membranes from lipid peroxidation, together with other functions (e.g., anti-inflammatory properties, stimulation of immune responses, and phagocytic functions) [5,24,25]. Toxic-element (As, Pb, Cd, and Hg)-driven effects on these vitamins in birds have been reported in different studies, as compiled in a recent review [5]. However, discrepancies have been found in the literature, and further studies are highly recommended to better understand the relation between exposure to metals and vitamin concentrations in different species, considering factors such as diet, age, and gender [5]. In this sense, to the best of our knowledge, this is the first study reporting 50 elements in blood and evaluating their potential effects on lutein and vitamin levels in the plasma of any *Turdidae* or *Columbidae* species.

Toxicological studies focused on widespread and common species are advantageous because they can be broadly used in standardized monitoring schemes over large regions, thus enabling comparisons among different countries, ecosystems, or environmental gradients. The Eurasian blackbird (*Turdus merula*) is a medium-sized, sedentary passerine with a wide distribution along the Palearctic region. Blackbirds originally inhabited woodlands and open forests, but have colonized a broad range of ecosystems in recent decades and are among the most common bird species in shrublands, farmlands, and urban areas [26,27]. In Spain, blackbirds occurs along all national territory, from coastal areas to 1500 m.a.s.l., being absent only in extremely arid, non-vegetated areas [28]. This species has an omnivorous diet, based mainly on ground-dwelling invertebrates, such as caterpillars, earthworms, snails, and fleshy fruits, when plentifully available [29,30]. Thus, the ubiquitous character and omnivorous diet, shown by the blackbird, together with their already reported ability to bioaccumulate contaminants [31], make them a potentially good model species to assess the exposure to toxic elements in highly impacted mining areas [32]. The rock pigeon (*Columba livia*) (hereafter, pigeon) is native to Eurasia and most countries of Africa, but it has been historically introduced around the world [33]. Its diet is mostly based on grain, though it may include other sources of protein, such as small invertebrates [34]. Pigeons often inhabit cities and rural areas but may be found in other ecosystems, such as farmlands and open forests, usually associated with man-made constructions (old farmhouses, bridges, and open-pit mines, among others). Therefore, the use of this species as a bioindicator of metal exposure can provide very useful information to assess the effects of mining activities in birds inhabiting human-dominated landscapes [35,36,37].

This study aims to evaluate blood concentrations of 50 elements (i.e., ATSDR’s list toxic elements, trace elements, REE, and ME) and their related effects on lutein and vitamin levels in blackbirds and pigeons inhabiting different scenarios of contaminant exposure in the southeast of the Iberian Peninsula (i.e., agricultural–urban area, mining area, and control area). We expect increased Pb, As, and other element concentrations in the mining area, based on previous findings in different wild bird species [16,17,38] that may lead to physiologic effects, including reduced hematocrit and vitamin alterations [16,18]. Thus, the potential effects of toxic element exposure on hematocrit levels and body measurements are also tested. 

## 2. Materials and Methods

### 2.1. Study Area and Pollution Scenarios

**Figure 1:**
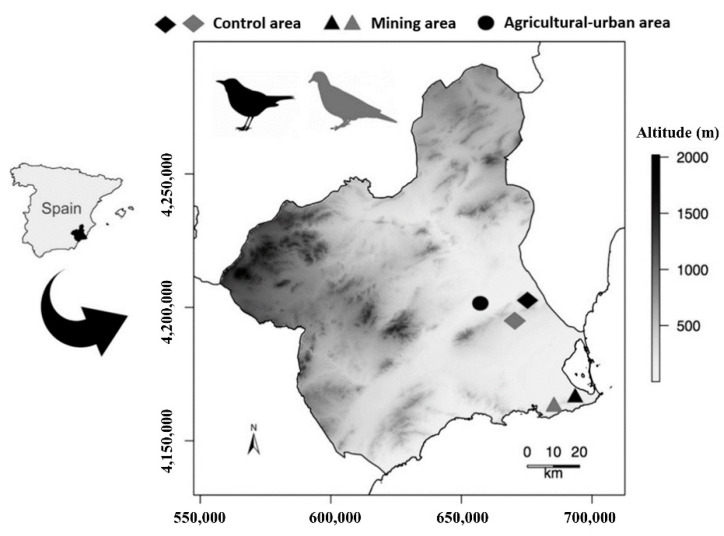
Map showing the investigated scenarios in the province of Murcia, Spain. The sampling areas are represented by a rhombus, triangle, and circle for control, mining, and agricultural–urban areas, respectively. The sampling areas for the different species are shown in different color: black figures for *Turdus merula* and grey figures for *Columba livia*.

The three pollution scenarios investigated were located in the province of Murcia, in the southeast of the Iberian Peninsula (37° 45′ N, 0° 57′ W) (Figure 1). This region is characterized by a Mediterranean semi-arid climate, with a strong water deficit during spring and summer and scarce rainfall occurring predominantly in winter. Irrigation agriculture is the main land use in lowlands and plateaus, whereas rainfed crops, pine forests, and Mediterranean shrublands extend over highlands and mountain areas. 

We selected three different scenarios of contaminant exposure in the study area, according to the main land use: agricultural–urban area (only for blackbird), mining area, and control area. The agricultural–urban area is placed in the center of the study province, on the outskirts of the city of Murcia (2 km away from the metropolitan area) and is represented by a mosaic of traditional tree farming (mostly citric crops) and scattered but moderately populated rural villages. Pesticides and other plant-protection agrochemicals are applied in this area through non-industrial methods (i.e., manual spraying). The mining area is represented by an ancient mining site (Cartagena-La Unión Mining District), placed in the south of the province of Murcia, where extraction activity was maintained until 1992 [39]. High contaminant exposure has been reported for wildlife inhabiting this area [40,41], even altering certain physiological functions, which could compromise long-term population viability [18]. To date, toxic metals are still spread from headwaters to lowlands during torrential rain episodes, thus impacting surrounding ecosystems [39]. In regards to the control area, it is located between the two other pollution scenarios and is represented by the Special Protection Area (SPA) “Sierras de Altaona y Escalona”, which is a mountainous zone dominated by rainfed traditional farming and Mediterranean forest. No contaminants have been previously found to affect wildlife in this area [38]. Human population density in this area is extremely low, with some scattered small rural villages in the territory, and the landscape is dominated by small tree crops (citrus, almond, and olive trees) scattered in a shrubland and forest matrix. 

### 2.2. Bird Sampling, Measurements, and Trace Element and Vitamin Analysis

Blackbirds (*n* = 42) were trapped by mist netting during the breeding season (June and July) of 2017 in the three described pollution scenarios. Nets (16-mm mesh size) were deployed from dawn to midmorning (7–12 am) beside vegetation, and conspecific playbacks were used, in order to maximize capture probabilities. Once captured, each bird was individually marked with a numbered aluminium ring (Ministerio de Medio Ambiente, ICONA, Madrid, Spain). Birds were aged and sexed on the basis of the available literature, by exploring moult limits on covert feathers and sexual dimorphism in plumage colour, respectively [42,43]. Except juveniles (young birds in their first summer), we were able to sex all trapped birds. We grouped birds into two age groups: juveniles (EURING age code 3) and breeding adults (birds in their second summer or older; EURING age code 4 or higher).

Pigeons (*n* = 27) were captured from March to May 2018 in the mining and control area. To trap the birds, mining open pits and old farmhouses were visited at night to locate wild pigeons on roosting and breeding sites. When detected, pigeons were caught by using a LED torch and handheld net with a 10 mm mesh-size [44]. Once captured, wing length and body mass were recorded for each bird and, whenever possible, they were aged and sexed, on the basis of the available literature [42]. Sex was difficult to determine, due to the great overlapping in body size and features between both sexes. Birds were also grouped into two age classes: juveniles (EURING age code 3) and breeding adults (EURING age code 4 or higher).

During handling and sampling for both species, best practice guidance was followed according to Espín et al. [45]. Individuals were examined prior to blood sampling, and all were considered clinically healthy. Blood samples (approximately 0.8 mL) were collected by puncturing the jugular (in blackbirds) and brachial (in pigeons) veins with 30G needles and 1 mL-syringes and stored in heparinized Eppendorf tubes under refrigerated conditions, until processed in the laboratory. One Eppendorf tube with whole blood was frozen at −80 °C until element analysis. Another Eppendorf tube with whole blood was centrifugated to separate plasma and red blood cell, and the tubes were frozen at −80 °C for biochemical analysis. The duration of the handling process per individual was ca. 15 min, and all birds were released exactly in the same location where they were caught. The sampling was approved by the Ethical Committee for Animal Experimentation at the University of Murcia (codes 193/2015 and 446/2018) and authorized by the “*Consejería de Agua, Agricultura y Medio Ambiente, Región de Murcia*” (AUF/2017/0039). 

A total of 50 elements (Table 1) were selected to be analyzed in whole blood, according to their toxicity and/or their frequent use in the manufacturing of electronic products [14,15]. Analyses were done using an Agilent 7900 ICP-MS equipment (Agilent Technologies, Santa Clara, CA, USA), following a procedure developed for human blood, which had been previously validated using certified reference materials [46]. Additional details were provided by Espín et al. [17]. Retinol, α-tocopherol, and lutein concentrations were measured in plasma samples by a HPLC-DAD-FLD system (Agilent 1200 Series), according to Rodríguez-Estival et al. [47]. Further information was provided in Espín et al. [18].

### 2.3. Statistical Procedures

Most of the elements showed some values below the limit of quantification (<LOQ), and for many of them, this proportion was relatively high (Table 1). For calculations, we substituted <LOQ values by a random number between 0 and LOQ [48]. For all 50 elements, we first calculated ranges and medians for both species (Table 1). For the statistical comparison, we only selected those elements (14 in *T. merula*, 11 in *C. livia*) where the proportion of <LOQ values was less than 20% [48] (Table 2). Three of those elements (Cu, Fe, and Mo) were normally distributed (by visual inspection of a histogram and Kolmogorov-Smirnov test for normality), while the rest of the elements were log_10_ transformed to make them better conform to normal distribution. Log_10_-transformation was also performed for the retinol and lutein values of *T. merula*, as well as for the tocopherol values of *C. livia*.

We analyzed differences in element levels, hematocrit, body measurements, and blood biochemistry among sampling sites, via linear models (LM) with area, sex, and age (only for *T. merula*) as explanatory factors. In most cases, sex and age showed no significant effects (*p* > 0.05), and site alone was left in the models as an explanatory factor to use with the full sample size (age and sex both had some missing values). However, sex was retained in the wing length models of both species, due to the significant sexual size dimorphism. For the values that showed significant differences among three sites (only for *T. merula*), we further ran Tukey’s test, adjusted for the number of pairwise comparisons. Alpha level was set to 0.05 in all analyses.

Finally, we selected major non-essential toxic trace elements, which showed increased values, relative to the control site (As, Cd, Hg, Mn, and Pb for *T. merula*; As, Cd and Pb for *C. livia*), for studying their associations to hematocrit and biochemical measures in plasma. Because these element levels were largely intercorrelated, a principal component analysis was performed to reduce the number of variables and to avoid collinearity problems in our models. For the consequent analyses, we included principal components that showed eigenvalues >1, i.e., PC1 and PC2, in *T. merula* (eigenvalues: PC1 2.5 and PC2 1.5; explaining 78% of variation) and PC1 in *C. livia* (eigenvalue: PC1 2.0; explaining 65% of variation). For both species, PC1 got positive loadings from all the elements and highest loadings from As, Cd, and Pb. In *T. merula*, PC2 got strong positive loading from Hg and negative loading from Mn. In the LMs, we used PCs, body mass, and wing length as explanatory factors for hematocrit and biochemical parameters. Model estimates and confidence limits for log_10_-transformed values were back-transformed to the original scale for tables and figures. 

Associations between blood elements, hematocrit (HT), plasma biochemistry, wing length, and mass were inspected by using the Pearson correlation test. All the analyses were run with SAS statistical software 9.4.

## 3. Results

In both bird species, most elements (especially REE and ME) in blood samples showed levels below LOQ in more than 20% of the individuals (Table 1), indicating generally low levels and suggesting limited toxic effects at the population level. For those elements where the proportion of <LOQ values was below 20%, mean concentrations by sampling area are shown in Table 2. *Turdus merula* showed increased levels of As, Cd, Pb, and Mn in the mining site, as compared to the control site, values of Pb being notably high (Table 2). Toxic elements As, Cd, and Pb were also increased in the agricultural–urban site, together with relatively high Hg values (Table 2). Trace elements Mo, Se, and Sr, instead, showed relatively low values in the mining site, although the level of Se was not significantly different from the control value (Table 2). Agricultural–urban site further showed lower Ba levels, as compared to the other sites (Table 2). Following a similar pattern, *C. livia* showed increased As, Cd, and Pb and decreased Sr levels in the mining site (Table 2). Finally, the levels of three toxic metals (As, Cd, and Pb) were directly compared between the two species within the mining site: As showed higher values in *C. livia* (F_1,25_ = 5.71, *p* = 0.025), Pb showed higher values in *T. merula* (F_1,25_ = 150, *p* < 0.0001), and Cd showed no difference (F_1,25_ = 0.12, *p* = 0.73).

Hematocrit, body mass, or wing length of *T. merula* were not dependent on the sampling site (Table 2), but males showed 4.8% longer wings than females (F_1,26_ = 23.4, *p* < 0.0001). Retinol levels were smaller in the agricultural–urban and mining site, as compared to the control (Table 2). The mining site also showed lower tocopherol and lutein levels than the other sites, but both measures differed significantly only from the agricultural–urban site (Table 2). None of the morphological or biochemical parameters showed significant between-site differences in *C. livia* (Table 2).

**Figure 2:**
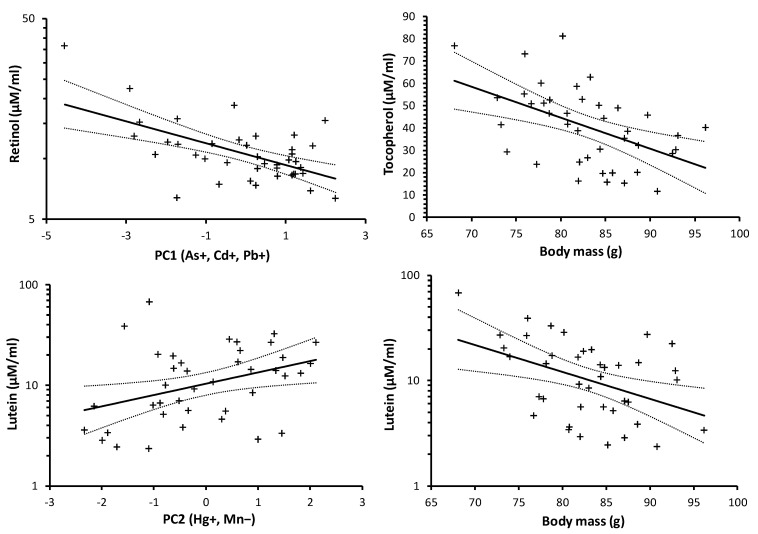
Biochemical markers in the plasma of *Turdus merula*, relative to the body mass and 1st and 2nd principal components (PC1 and PC2), describing the levels of toxic metals in blood (directions for the main factor loadings are shown in parentheses). Prediction line and 95% confidence limits come from the models of Table 3 (log-transformed values transformed back to the original scale). Plus signs denote the original data points.

Hematocrit showed a positive relationship with PC2 _(Hg+, Mn−)_ in *T. merula* and PC1 _(As+, Cd+, Pb+)_ in *C. livia*, but both effects were only marginally significant (*p* = 0.051 and *p* = 0.075, respectively; Table 3). In *T. merula*, retinol was negatively associated with PC1 _(As+, Cd+, Pb+)_ and lutein was positively associated with PC2 _(Hg+, Mn−)_ (Table 3 and Figure 2). Tocopherol and lutein levels further decreased with increasing body mass (Table 3 and Figure 2). In *C. livia*, none of the biochemical parameters were associated with PC1 _(As+, Cd+, Pb+)_ or morphological measures (Table 3). Correlations among all parameters are shown for both species in Table A1 (Appendix A).

## 4. Discussion

### 4.1. Element Concentrations in Blood

**Figure 3:**
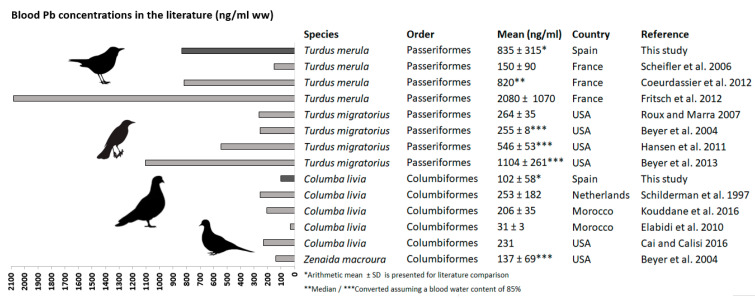
Blood Pb concentrations (ng/mL, w.w.) reported in Passeriformes and Columbiformes inhabiting polluted environments.

Blood concentrations of As, Cd, and Pb (and Mn in *T. merula*) were increased in both *T. merula* and *C. livia* captured in the mining area, compared to the control site. The higher exposure to the most toxic elements for both species in the ancient mine site was expected. This mining area has been exploited for more than 2500 years (mainly for Pb, Zn, Cu, Mn, Ag, Fe, and Sn extraction) [49,50], and similar results have already been observed in several biomonitoring studies developed along the years (1993–2020) in other bird species, including red-necked nightjars (*Caprimulgus ruficollis*) and eagle owls (*Bubo bubo*) [16,17,38,40,51,52]. The Pb concentrations detected in blood of *T. merula* in the mining area are of special concern, being 58 times higher than the levels found in birds from the control area. These Pb levels (arithmetic mean = 835 ng/mL w.w.) are indicative of a high level of exposure and are similar to [53,54] or higher than those reported in *T. merula* and *T. migratorius* in polluted environments in France and USA, respectively [31,55,56,57] (Figure 3). On the other hand, *C. livia* showed blood Pb levels (arithmetic mean = 102 ng/mL w.w.) similar to or lower than those reported in *C. livia* and *Zenaida macroura* in polluted environments in the Netherlands, Morocco, and USA [56,58,59,60] (Figure 3).

Regarding As blood concentrations in *T. merula* and *C. livia* in the mining area (arithmetic means = 6.7 and 29.3 ng/mL w.w., respectively), *T. merula* showed similar levels, but *C. livia* showed higher levels than those reported in red-winged blackbirds (*Agelaius phoeniceus*) and marsh wrens (*Cistothorus palustris*) in the USA (4.64 and 3.73 ng/mL) [61]. The concentrations of As and Pb were compared between the two species within the mining site, and Pb values were higher in *T. merula*, while As showed higher values in *C. livia*. These results could be related to their different diets. *T. merula* has an omnivorous diet, based mainly on ground-dwelling invertebrates and fleshy fruits (when plentifully available), while the diet of *C. livia* is grain-based [29,30,34]. In this line, previous studies have shown that soil-dwelling invertebrates are more likely to accumulate Pb than plants [62], and birds consuming omnivorous and invertebrate diets show higher Pb concentrations than granivore species [63]. On the other hand, As uptake by plants will depend on its concentration and speciation in soils, and plants growing in contaminated sites have shown high As levels (1.14–98.5 mg/kg), compared to uncontaminated areas (0.06–0.58 mg/kg) [64]. A study analyzing soil samples and plants, naturally growing in our study’s mining site, showed high concentrations of As in soil (mean = 860 mg/kg), which correlated with As levels in plant leaves (mean = 23.5 mg/kg) [65]. Different plants for human and animal consumption have been reported to accumulate As concentrations in grain, representing their edible tissues an important source of dietary As in humans [66,67]. Therefore, the mostly grain-based diet of *C. livia* could explain the higher blood As concentrations in this species, compared to *T. merula*. In addition, different detoxication mechanisms for As can also explain these results, since methylating capacity is highly dependent on species [2], and a lower dietary content of methionine or protein can also result in a lower As methylation [68].

Concentrations of Cd in *T. merula* and *C. livia* in the mining area (arithmetic means = 0.67 and 0.55 ng/mL w.w., respectively) were lower than those found in *Agelaius phoeniceus, Cistothorus palustris*, and tree swallows (*Tachycineta bicolor*) in the USA (13.5, 26.9, and 3.58 ng/mL, respectively) [61], and these levels are considered low in birds [17,38,51,61]. Mn concentrations in *T. merula* (arithmetic mean = 23.4 ng/mL w.w.) in the mining site were similar to the levels observed in Northwestern crows (*Corvus caurinus*) (28–33 ng/mL w.w.) in the USA [69].

On the other hand, the blood levels of Mo and Se (only in *T. merula*), as well as Sr (in both species), were lower in the mining area. Pollutant-related indirect effects, including poor quality and quantity of food or changes in the diet (due to resource limitations), may contribute to lower essential elements (Mo and Se) and Sr concentrations in the mining-impacted area. Previous studies have also found reduced Mo and Sr levels in red-necked nightjars and eagle owls inhabiting the mining area [16,17]. Although Sr is classically considered a non-essential element, further studies are needed to understand its essentiality, since its supplementation has been shown to increase calcified bone volume and limit bone resorption, which prevents from bone mass loss [70,71,72].

*T. merula* also showed increased As, Cd, and Pb concentrations in blood in the agricultural–urban area, as compared to the control area, together with the highest Hg levels. Contamination sources in this agricultural–urban area could include agricultural practices and industrial activities [73]. Individuals from this area also showed the highest Se concentrations. The accumulation of Se in tissues under Hg exposure has been well documented, since, at proper concentrations, Se can form a complex with Hg and protects against its toxicity [74,75]. In the present study, a significant positive correlation was found between Se and Hg levels in blood in *T. merula* (r = 0.62; *p* < 0.001; Table A1).

### 4.2. Element-Driven Effects on Lutein and Vitamin Levels, as well as Hematocrit and Body Measurements 

Blood Pb concentrations in *T. merula* inhabiting the mining area were within the range related to subclinical signs, with 43% of birds exceeding the benchmark value for physiological effects in Anseriformes and Falconiformes (i.e., 200 ng/mL w.w.), 33% exceeding the level considered of clinical poisoning (i.e., 500 ng/mL), and 14% (6 individuals) exceeding the threshold related to severe clinical poisoning in Anseriformes and Falconiformes (i.e., 1000 ng/mL) [76]. Although it is well known that different avian species may have different sensitivity to metals, the blood Pb concentrations achieved in individuals from the mining area may have important consequences to their heath. In this sense, eagle owls living in the same mining site showed up to a 79% inhibition of δALAD in blood at Pb concentrations above 190 ng/mL [77], red-necked nightjars showed a 4–44% decrease in hematocrit values at blood concentrations ranging 200–>1000 ng/mL [17], and the blood Pb levels of 30 and 150 ng/mL in eagle owls and griffon vultures (*Gyps fulvus*), respectively, depleted the levels of different antioxidants in red blood cells and induced lipid damage in erythrocytes [38,78]. These concentrations could have also adversely affect bird reproduction; e.g., experimentally-dosed red-legged partridges (*Alectoris rufa*) showed a lower hatching rate, reduced sperm motility, and acrosome integrity at blood Pb levels of 758 and 920 ng/mL in females and males, respectively [79].

In this study, a decrease of 33 and 38% in the plasma retinol levels in *T. merula* inhabiting the mining and the agricultural–urban areas, respectively, as compared to the control site, was associated with increased Pb, As, and Cd exposure. This could be due to a metal-driven suppressive effect in retinol storage and metabolism [5]. In line with this, it has been suggested that metals can disrupt the expression of genes involved in retinoid homeostasis and interfere with proteins/enzymes involved in its transport, esterification, and hydrolysis [5,80]. In addition, as retinol also functions as antioxidant, an over-use of this molecule to cope with metal-related oxidative stress could also explain this result [5]. This metal-driven decrease tendency in retinol levels is consistent with the results observed by other authors in different bird species exposed to Pb, As, Cd, and Hg in both field and experimental studies [18,81,82,83,84,85], and it could have adverse consequences to birds’ growth and development [5].

In spite of the direct effects of Pb, As, and Cd on retinol levels, such direct effects were not found in tocopherol and lutein concentrations in plasma. The decreased levels of tocopherol and lutein in *T. merula* in the mining area, compared to the agricultural–urban area (decrease of 33 and 58%, respectively; significant difference) and the control site (decrease of 30 and 48%, respectively; non-significant difference), could be related to differences in diet among the study areas. Birds probably have access to nutrient-rich items in the agricultural–urban and the control sites, while the diet in the mining area could be poorer in tocopherol and lutein, due to metal-related indirect effects. In this sense, plasma tocopherol and lutein were positively correlated (r = 0.68, *p* < 0.001; Table A1), and they were inversely related to body mass (Figure 2). Birds in better conditions (with larger fat stores) could show lower circulating carotenoids because they get stored in fat tissue [86]. Moreover, it has been reported that lutein supplementation increased the birds’ fat reserves, possibly indicating that carotenoids interfere with the lipid metabolism [87]. Previous studies have observed that metal-polluted environments negatively affect lutein levels in different bird species, due to lower access to carotenoid-rich diet [8,85]. In addition, Mn concentrations in *T. merula* are negatively associated to lutein levels, thus increased blood Mn in the mining area could have produced a decrease in plasma lutein levels. Excessive Mn may increase free radicals, and fighting against them would again deplete antioxidants, such as lutein, in the body. Mn concentrations (and the effect on lutein levels in birds) have rarely been reported [16,18]. However, Mn toxicity on pigment content, including decreased carotenoid levels, has been found in different plant species [88,89].

Despite *C. livia* showing increased As, Cd, and Pb concentrations in the mining area; the levels of retinol, tocopherol, and lutein in plasma did not differ between study sites and were not affected by metals. Only blood As concentrations were positively correlated with tocopherol levels, which could be related to a protective response (as tocopherol is a potent antioxidant) to cope with As-related oxidative stress [5]. Interspecific differences in the metabolism of vitamins/carotenoids, or in the tolerance to metals, could be the reason underlying these contradictory results. In this regard, results from previous studies suggest that some species can be less susceptible to metal-related disturbances in physiological parameters [8,38,77,78,83,85]. This could be related to among-species variations in regulation mechanisms and vitamin upregulation thresholds [5]. Moreover, the smaller sample size, the lower the Pb concentrations in blood and the fact that pigeons could not be sampled from the agricultural–urban area may partly explain the lack of effects of metals on physiology in this species.

## 5. Conclusions

Local contamination in the mining area contributes to increased concentrations of As, Cd, and Pb (and Mn in *T. merula*) in the blood of *T. merula* and *C. livia*, while potential differences in food quality and quantity in that environment may account for the decreased blood Sr in both species, as well as the decreased blood Mo, Se, plasma tocopherol, and lutein in *T. merula*.

Hematocrit and morphological parameters were unaffected by the mining-impacted environment. However, increased Pb, As, and Cd exposure were associated with decreased plasma retinol levels in the *T. merula* inhabiting the mining and agricultural–urban areas (33 and 38% decrease, respectively), as compared to the control site. This could be due to a metal-driven, suppressive effect in retinol storage and metabolism, and/or an over-use of retinol, to prevent metal-related oxidative stress. This retinol depletion could have adverse consequences in the growth and development for this species. 

According to the results found in this study, *C. livia* could be less susceptible to metals (none of the biochemical parameters were associated to toxic metals) than *T. merula*. However, additional studies evaluating other biochemical parameters and with higher number of samples are needed to support these findings.

## Figures and Tables

**Table 1:** Ranges and medians for element concentrations ^a^ (ng/mL, w.w.) in whole blood of the Eurasian blackbird (*Turdus merula*) and wild rock pigeon (*Columba livia*). Limits of quantification (LOQ) given together with the proportion of samples <LOQ.

Element	Group ^b^	LOQ	*T. merula* (*n* = 42)			*C. livia* (*n* = 27)		
			Range (min—max)	Median	%<LOQ	Range (min—max)	Median	%<LOQ
*Aluminum (Al)*	2	38.426	<LOQ—2010	16.9	95	<LOQ—365	15.0	96
*Antimony (Sb)*	2	0.010	<LOQ—7.04	0.0060	95	N/A	<LOQ	100
*Arsenic (As)*	2	0.008	<LOQ—44.2	2.56	2	0.686—72.2	2.33	0
*Barium (Ba)*	2	1.016	4.64—44.2	15.0	0	<LOQ—56.4	7.12	44
*Beryllium (Be)*	2	0.050	<LOQ—0.597	0.0208	88	<LOQ—0.543	0.0317	96
*Bismuth (Bi)*	4	0.050	<LOQ—0.464	0.0204	95	N/A	<LOQ	100
*Cadmium (Cd)*	2	0.010	<LOQ—2.61	0.254	19	<LOQ—1.08	0.328	19
*Cerium (Ce)*	3	0.050	<LOQ—1.92	0.554	40	<LOQ—1.12	0.0258	96
*Chromium (Cr)*	1, 2	0.229	<LOQ—12.3	1.10	12	<LOQ—11.3	0.138	81
*Cobalt (Co)*	1, 2	0.011	0.350—2.72	1.01	0	<LOQ—1.52	0.821	30
*Copper (Cu)*	1, 2	1.724	137—310	200	0	153—299	225	0
*Dysprosium (Dy)*	3	0.005	<LOQ—0.0409	0.0027	88	<LOQ—0.0294	0.0026	89
*Erbium (Er)*	3	0.050	N/A	<LOQ	100	N/A	<LOQ	100
*Europium (Eu)*	3	0.005	<LOQ—0.0122	0.0035	71	<LOQ—0.0103	0.0019	96
*Gadolinium (Gd)*	3	0.005	<LOQ—0.0523	0.0036	62	<LOQ—0.0610	0.0027	93
*Gallium (Ga)*	4	0.050	<LOQ—0.365	0.127	29	<LOQ—0.292	0.173	33
*Gold (Au)*	4	0.005	<LOQ—2.08	0.0025	95	N/A	<LOQ	100
*Holmium (Ho)*	3	0.005	<LOQ—0.0064	0.0024	95	<LOQ—0.0061	0.0030	93
*Indium (In)*	4	0.005	<LOQ—0.0402	0.0035	60	<LOQ—0.0419	0.0027	85
*Iron (Fe)*	1	24.645	246,000—410,000	330,000	0	221,000—476,000	400,000	0
*Lanthanum (La)*	3	0.100	<LOQ—0.285	0.0539	81	<LOQ—0.591	0.0435	96
*Lead (Pb)*	2	0.361	5.36—1350	114	0	<LOQ—252	30.6	7
*Lutetium (Lu)*	3	0.005	N/A	<LOQ	100	N/A	<LOQ	100
*Manganese (Mn)*	1, 2	0.371	7.84—53.8	16.8	0	10.5—94.9	17.9	0
*Mercury (Hg)*	2	0.010	4.34—197	33.7	0	<LOQ—24.4	0.0070	74
*Molybdenum (Mo)*	1	0.148	3.23—40.6	18.1	0	<LOQ—29.1	14.1	4
*Neodymium (Nd)*	3	0.005	<LOQ—0.282	0.0023	83	N/A	<LOQ	100
*Nickel (Ni)*	1, 2	7.946	N/A	<LOQ	100	<LOQ—158	4.18	93
*Niobium (Nb)*	4	0.005	<LOQ—0.256	0.0032	76	<LOQ—0.320	0.0025	96
*Osmium (Os)*	4	0.005	N/A	<LOQ	100	<LOQ—0.0059	0.0030	70
*Palladium (Pd)*	2	0.010	N/A	<LOQ	100	<LOQ—0.0611	0.0050	74
*Platinum (Pt)*	4	0.005	N/A	<LOQ	100	<LOQ—0.0772	0.0047	56
*Praseodymium (Pr)*	3	0.005	<LOQ—0.0798	0.0029	90	N/A	<LOQ	100
*Ruthenium (Ru)*	4	0.005	<LOQ—0.0114	0.0031	95	<LOQ—0.0111	0.0025	89
*Samarium (Sm)*	3	0.005	<LOQ—0.0626	0.0027	95	<LOQ—0.0491	0.0020	96
*Selenium (Se)*	1, 2	0.153	93.6—10,700	520	0	139—621	330	0
*Silver (Ag)*	2	0.100	<LOQ—3.09	0.0833	64	<LOQ—0.697	0.0393	89
*Strontium (Sr)* ^c^	2	0.439	28.5—162	58.7	0	22.4—153	49.5	0
*Tantalum (Ta)*	4	0.005	<LOQ—0.219	0.0047	60	N/A	<LOQ	100
*Terbium (Tb)*	3	0.005	N/A	<LOQ	100	N/A	<LOQ	100
*Thallium (Tl)*	2	0.050	<LOQ—0.914	0.0750	33	<LOQ—0.748	0.0456	59
*Thorium (Th)*	2	0.050	<LOQ—0.0599	0.0281	93	<LOQ—0.182	0.0329	93
*Thulium (Tm)*	3	0.005	N/A	<LOQ	100	N/A	<LOQ	100
*Tin (Sn)*	2	0.010	<LOQ—5.42	0.170	45	<LOQ—2.74	0.0051	85
*Titanium (Ti)*	4	0.757	<LOQ—23.4	14.5	43	<LOQ—62.6	0.521	67
*Uranium (U)*	2	0.050	N/A	<LOQ	100	<LOQ—0.0785	0.0302	96
*Vanadium (V)*	2	0.050	<LOQ—1.29	0.0358	67	<LOQ—8.41	2.20	7
*Ytterbium (Yb)*	3	0.005	<LOQ—0.0147	0.0020	76	<LOQ—0.0103	0.0034	78
*Yttrium (Y)*	3	0.005	<LOQ—0.195	0.0038	62	<LOQ—0.198	0.0026	96
*Zinc (Zn)*	1, 2	51.031	4030—5980	4950	0	2190—7320	5600	0

^a^ For calculating medians, <LOQ values were replaced by a random number between 0 and LOQ. ^b^ Element categories: 1 = Essential trace elements, 2 = ATSDR’s list toxic elements, 3 = Rare earth elements, and 4 = Other minor elements. ^c^ Stable Sr is considered to be of relatively low toxicity, and only Sr-90 is included in the ATSDR’s Substance Priority List.

**Table 2:** Mean (±95% confidence limits) concentrations (ng/mL, w.w.) of the elements in the blood, hematocrit, body measurements, and plasma biochemistry of the Eurasian blackbird (*Turdus merula*; *n* = 42) and wild rock pigeon (*Columba livia; n* = 27) in the agricultural–urban area, mining area, and control area. Linear models (LM) for comparison of means. Tukey’s test: means with the same letter are not statistically different.

		Mean			LM	
***T. merula***	**Variable**	**Agric.-urban (CL)**	**Mining (CL)**	**Control (CL)**	**F _df_**	***p***
	*Arsenic (As)* *	2.87 ^a^ (1.34–6.15)	3.81 ^a^ (2.01–7.21)	0.494 ^b^ (0.238–1.03)	9.98 _2,39_	**0.0003**
	*Barium (Ba)* *	8.81 ^a^ (6.57–11.8)	17.6 ^b^ (13.7–22.5)	16.5 ^b^ (12.5–21.9)	7.57 _2,39_	**0.0017**
	*Cadmium (Cd)* *	0.185 ^a^ (0.0722–0.474)	0.523 ^a^ (0.237–1.15)	0.0147 ^b^ (0.00597–0.0364)	18.5 _2,39_	**<0.0001**
	*Chromium (Cr)* *	0.716 (0.362–1.41)	1.28 (0.720–2.26)	0.650 (0.338–1.25)	1.49 _2,39_	0.24
	*Cobalt (Co)* *	1.04 (0.809–1.33)	1.15 (0.931–1.41)	0.808 (0.637–1.03)	2.56 _2,39_	0.090
	*Copper (Cu)*	219 (198–239)	206 (188–223)	188 (168–208)	2.46 _2,39_	0.099
	*Iron (Fe)*	336,000 (310,000–363,000)	327,000 (304,000–349,000)	336,000 (310,000–361,000)	0.22 _2,39_	0.81
	*Lead (Pb)* *	109 ^a^ (80.4–148)	779 ^b^ (603–1010)	13.4 ^c^ (9.98–17.9)	224 _2,39_	**<0.0001**
	*Manganese (Mn)* *	14.8 ^a^ (12.0–18.3)	21.9 ^b^ (18.3–26.1)	14.9 ^a^ (12.1–18.2)	5.79 _2,39_	**0.0063**
	*Mercury (Hg)* *	77.5 ^a^ (46.0–131)	21.5 ^b^ (13.9–33.4)	29.6 ^b^ (17.9–48.8)	7.49 _2,39_	**0.0018**
	*Molybdenum (Mo)*	26.6 ^a^ (23.5–29.7)	11.1 ^b^ (8.47–13.7)	19.8 ^c^ (16.8–22.8)	30.5 _2,39_	**<0.0001**
	*Selenium (Se)* *	1020 ^a^ (578–1810)	415 ^b^ (257–671)	788 ^ab^ (455–1360)	3.32 _2,39_	**0.047**
	*Strontium (Sr)* *	66.1 ^a^ (55.7–78.4)	50.2 ^b^ (43.5–58.0)	79.7 ^a^ (67.7–94.0)	9.46 _2,39_	**0.0004**
	*Zinc (Zn)* *	5040 (4760–5340)	4940 (4710–5190)	4820 (4560–5090)	0.69 _2,39_	0.51
	*Hematocrit* (%)	41.9 (38.2–45.5)	37.2 (34.2–40.3)	39.1 (35.6–42.6)	1.96 _2,39_	0.15
	*Body mass* (g)	82.8 (79.3–86.3)	84.5 (81.6–87.5)	80.9 (77.6–84.3)	1.31 _2,39_	0.28
	*Wing length* (mm) **	122 (120–124)	124 (123–126)	123 (120–125)	1.55 _2,26_	0.23
	*Retinol* (µM/mL) *	8.92 ^a^ (7.59–10.5)	9.54 ^a^ (8.26–11.0)	14.3 ^b^ (12.3–16.7)	11.3 _2,37_	**0.0002**
	*Tocopherol* (µM/mL)	47.3 ^a^ (37.9–56.8)	31.7 ^b^ (23.2–40.1)	45.0 ^ab^ (35.9–54.1)	3.79 _2,37_	**0.032**
	*Lutein* (µM/mL) *	15.1 ^a^ (9.55–23.9)	6.40 ^b^ (4.24–9.66)	12.2 ^ab^ (7.82–18.9)	4.47 _2,37_	**0.018**
***C. livia***	**Variable**	**Agric.-urban (CL)**	**Mining (CL)**	**Control (CL)**	**F _df_**	***p***
	*Arsenic (As)* *	N/A	12.5 (6.47–24.2)	1.93 (1.16–3.20)	21.4 _1,25_	**<0.0001**
	*Cadmium (Cd)* *	N/A	0.478 (0.145–1.58)	0.0799 (0.0320–0.200)	5.99 _1,25_	**0.022**
	*Copper (Cu)*	N/A	224 (199–249)	215 (196–235)	0.31 _1,25_	0.58
	*Iron (Fe)*	N/A	408,000 (371,000–446,000)	391,000 (362,000–420,000)	0.58 _1,25_	0.45
	*Lead (Pb)* *	N/A	90.6 (35.8–229)	9.02 (4.43–18.4)	16.5 _1,25_	**0.0004**
	*Manganese (Mn)* *	N/A	23.0 (16.0–33.2)	18.9 (14.3–25.0)	0.78 _1,25_	0.39
	*Molybdenum (Mo)*	N/A	15.9 (11.9–19.8)	13.1 (10.0–16.1)	1.34 _1,25_	0.26
	*Selenium (Se)* *	N/A	397 (322–490)	312 (266–366)	3.58 _1,25_	0.070
	*Strontium (Sr)* *	N/A	38.8 (29.3–51.5)	59.3 (47.7–73.6)	5.96 _1,25_	**0.022**
	*Vanadium (V)* *	N/A	2.34 (0.985–5.54)	1.28 (0.661–2.48)	1.29 _1,25_	0.27
	*Zinc (Zn)* *	N/A	5400 (4610–6330)	5170 (4580–5850)	0.20 _1,25_	0.66
	*Hematocrit* (%) ***	N/A	64.0 (55.7–72.3)	57.6 (54.1–61.1)	2.25 _1,18_	0.15
	*Body mass* (g)	N/A	341 (311–371)	330 (307–353)	1.25 _1,25_	0.42
	*Wing length* (mm) **	N/A	233 (229–237)	230 (226–234)	1.20 _1,13_	0.29
	*Retinol* (µM/mL)	N/A	7.96 (6.17–9.74)	9.53 (8.17–10.9)	2.09 _1,25_	0.16
	*Tocopherol* (µM/mL) *	N/A	32.5 (21.2–49.9)	33.0 (23.8–45.8)	0.00 _1,25_	0.95
	*Lutein* (µM/mL)	N/A	10.5 (4.88–16.2)	13.9 (9.58–18.2)	0.96 _1,25_	0.34

* Geometric means, values log_10_ transformed for the analyses and back-transformed for the table. ** Sex was included as an explanatory variable in the model and 12 individuals of *T. merula* and 11 individuals of *C. livia* could not be determined for sex, decreasing the sample size. *** There were seven missing hematocrit values in *C. livia*.

**Table 3:** Linear models for explaining variation in hematocrit value and the biochemical plasma variables of *Turdus merula* and *Columba livia*. Principal components (PC1 and PC2) describe variations in the concentration of major toxic metals in blood (see methods). Statistically significant effects are shown in bold.

		F (Estimate ± SE)			
*T. merula*	*n*	PC1 _(As+, Cd+, Pb+)_	PC2 _(Hg+, Mn−)_	Body mass	Wing length
Hematocrit (%)	42	0.08 (0.19 ± 0.65)	4.07 (1.62 ± 0.80) °	0.00 (−0.01 ± 0.17)	2.18 (−0.37 ± 0.25)
Retinol (μM/mL) ^a^	40	**14.1 (−0.052 ± 0.014**) ***	0.65 (−0.013 ± 0.016)	0.20 (−0.0016 ± 0.0035)	0.01 (0.00058 ± 0.0053)
Tocopherol (μM/mL)	40	0.79 (−1.52 ± 1.70)	1.39 (2.37 ± 2.01)	**8.85 (−1.26 ± 0.42)** **	0.00 (−0.038 ± 0.64)
Lutein (μM/mL) ^a^	40	0.22 (−0.017 ± 0.035)	**7.84 (0.12 ± 0.041)** **	**7.18 (−0.023 ± 0.087)** *	0.67 (−0.011 ± 0.013)
*C. livia*	*n*	PC1 _(As+, Cd+, Pb+)_		Body mass	Wing length
Hematocrit	20	3.82 (2.05 ± 1.05) °		0.00 (0.00056 ± 0.041)	2.63 (0.33 ± 0.20)
Retinol (μM/mL)	27	0.32 (−0.23 ± 0.40)		0.05 (0.0036 ± 0.017)	2.02 (0.12 ± 0.084)
Tocopherol (μM/mL) ^a^	27	1.07 (0.043 ± 0.042)		0.08 (0.00050 ± 0.0017)	1.51 (−0.011 ± 0.0086)
Lutein (μM/mL)	27	0.02 (−0.21 ± 1.36)		0.05 (−0.012 ± 0.057)	0.27 (0.15 ± 0.28)

^a^ Variable was log_10_ transformed for the analysis. ° *p* < 0.1, * *p* < 0.05, ** *p* < 0.01, *** *p* < 0.0001.

## Data Availability

The data presented in this study are available on request from the corresponding author.

## Appendix A

**Table A1:** Pearson correlations between blood elements, hematocrit (HT), plasma biochemistry, wing length, and body mass of *Turdus merula* (above diagonal, *n* = 42; for retinol, tocopherol, and lutein *n* = 40) and *Columba livia* (below diagonal, *n* = 27; for hematocrit *n* = 20).

	As	Ba	Cd	Co	Cr	Cu	Fe	Hg	Mn	Mo	Pb	Se	Sr	V	Zn	HT	Ret	Toc	Lut	Mass	Wing
As		−0.062	**0.69 *****	**0.65 *****	0.18	0.27	0.27	**0.42 ****	0.062	−0.20	**0.54 *****	**0.47 ****	−0.19	N/A	0.11	0.028	**−0.54 *****	−0.22	−0.10	0.25	0.29
Ba	N/A		−0.072	0.056	0.13	0.086	−0.26	−0.28	**0.46 ****	−0.13	0.12	−0.12	**0.39 ***	N/A	0.069	−0.29	0.28	−0.13	−0.17	−0.14	−0.15
Cd	**0.42 ***	N/A		**0.50 *****	0.26	0.031	0.0035	0.25	0.23	**−0.34 ***	**0.71 *****	0.20	**−0.34 ***	N/A	−0.0024	−0.011	**−0.48 ****	−0.25	−0.18	**0.36 ***	0.25
Co	N/A	N/A	N/A		**0.41 ****	0.18	0.013	**0.39 ***	0.25	−0.22	0.29	**0.42 ****	−0.034	N/A	0.25	−0.14	−0.20	−0.20	0.0017	0.19	0.15
Cr	N/A	N/A	N/A	N/A		0.17	0.071	0.092	0.19	−0.24	0.11	0.12	−0.11	N/A	0.22	−0.25	−0.012	−0.28	0.040	0.14	0.21
Cu	0.30	N/A	0.29	N/A	N/A		0.30	0.16	0.052	0.010	0.23	−0.056	0.11	N/A	0.22	0.11	−0.22	0.036	−0.022	0.018	−0.041
Fe	0.37	N/A	**0.42 ***	N/A	N/A	**0.63 *****		0.16	−0.23	−0.21	−0.090	0.26	−0.14	N/A	0.30	**0.58 *****	−0.059	0.28	**0.31 ***	−0.063	−0.012
Hg	N/A	N/A	N/A	N/A	N/A	N/A	N/A		−0.21	**0.41 ****	−0.15	**0.62 *****	0.17	N/A	0.084	0.29	−0.17	0.12	0.30	0.12	−0.17
Mn	0.16	N/A	0.21	N/A	N/A	0.34	0.28	N/A		−0.15	**0.44 ****	−0.30	−0.075	N/A	0.20	−0.25	−0.063	−0.21	**−0.33 ***	0.11	−0.13
Mo	0.38	N/A	0.21	N/A	N/A	**0.50 ****	**0.53 ****	N/A	0.35		**−0.43 ****	0.26	**0.39 ***	N/A	0.061	0.16	0.10	0.25	0.28	−0.22	−0.29
Pb	**0.56 ****	N/A	**0.39 ***	N/A	N/A	0.17	**0.46 ***	N/A	0.16	**0.48 ***		−0.27	**−0.51 *****	N/A	0.13	−0.064	**−0.47 ****	−0.25	**−0.34 ***	0.26	0.24
Se	−0.054	N/A	0.17	N/A	N/A	0.37	**0.49 ****	N/A	0.14	**0.40 ***	**0.39 ***		0.27	N/A	0.18	0.062	−0.0013	0.046	0.28	0.12	0.18
Sr	−0.034	N/A	−0.044	N/A	N/A	**0.58 ****	0.36	N/A	**0.50 ****	**0.42 ***	−0.14	−0.029		N/A	−0.0032	−0.088	0.13	−0.013	−0.048	−0.16	−0.24
V	0.35	N/A	0.28	N/A	N/A	0.19	0.37	N/A	0.075	**0.56 ****	**0.54 ****	0.29	0.14		N/A	N/A	N/A	N/A	N/A	N/A	N/A
Zn	0.19	N/A	**0.41 ***	N/A	N/A	**0.61 *****	**0.86 *****	N/A	0.36	**0.59 ****	**0.42 ***	**0.51 ****	**0.42 ***	**0.50 ****		0.19	0.17	0.25	0.22	−0.13	0.054
HT	0.20	N/A	**0.55 ***	N/A	N/A	0.24	**0.65 ****	N/A	0.19	0.088	0.22	**0.47 ***	−0.22	0.17	**0.61 ****		−0.048	0.29	0.21	−0.034	−0.23
Ret	−0.29	N/A	0.23	N/A	N/A	−0.094	0.20	N/A	−0.24	−0.17	0.038	0.26	−0.17	0.062	0.26	**0.61 ****		**0.47 ****	**0.39 ***	−0.26	−0.20
Toc	**0.42 ***	N/A	0.0023	N/A	N/A	0.26	0.29	N/A	0.35	0.21	0.013	−0.25	0.19	−0.030	0.10	0.26	−0.13		**0.68 *****	**−0.50 ****	−0.15
Lut	0.16	N/A	−0.14	N/A	N/A	−0.032	0.15	N/A	−0.027	−0.0090	−0.030	−0.20	0.023	0.027	0.15	0.10	0.31	**0.54 ****		**−0.43 ****	−0.22
Mass	0.12	N/A	**0.52 ****	N/A	N/A	−0.025	0.32	N/A	0.082	−0.073	0.12	0.12	−0.063	0.26	**0.41 ***	0.15	0.28	−0.072	0.025		0.20
Wing	0.033	N/A	0.30	N/A	N/A	−0.27	0.13	N/A	−0.18	−0.32	0.063	0.12	**−0.39 ***	0.17	0.16	**0.41 ***	**0.40 ***	−0.24	0.098	**0.68 *****	

HTC = hematocrit (%), Ret = retinol in plasma (µM/mL), Toc = tocopherol in plasma (µM/mL), Lut = lutein in plasma (µM/mL), Wing = wing length (mm), Mass = body mass (g). As, Ba, Cd, Co, Cr, Hg, Mn, Mo, Pb, Se, Sr, Zn, RET, and LUT were log_10_-transformed for the analysis in *T. merula*. As, Ba, Cd, Co, Cr, Hg, Mn, Mo, Pb, Se, Sr, V, Zn, and TOC were log_10_-transformed for the analysis in *C. livia*; * *p* < 0.05, ** *p* < 0.01, *** *p* < 0.001. N/A = not applicable.
